# Expression of Tra2****β**** in Cancer Cells as a Potential Contributory Factor to Neoplasia and Metastasis

**DOI:** 10.1155/2013/843781

**Published:** 2013-07-08

**Authors:** Andrew Best, Caroline Dagliesh, Ingrid Ehrmann, Mahsa Kheirollahi-Kouhestani, Alison Tyson-Capper, David J. Elliott

**Affiliations:** ^1^Institute of Genetic Medicine, Newcastle University, Central Parkway, Newcastle upon Tyne NE1 3BZ, UK; ^2^Institute of Cellular Medicine, Newcastle University, Framlington Place, Newcastle upon Tyne NE2 4HH, UK

## Abstract

The splicing regulator proteins SRSF1 (also known as ASF/SF2) and SRSF3 (also known as SRP20) belong to the SR family of proteins and can be upregulated in cancer. The *SRSF1* gene itself is amplified in some cancer cells, and cancer-associated changes in the expression of *MYC* also increase *SRSF1* gene expression. Increased concentrations of SRSF1 protein promote prooncogenic splicing patterns of a number of key regulators of cell growth. Here, we review the evidence that upregulation of the SR-related Tra2**β** protein might have a similar role in cancer cells. The *TRA2B* gene encoding Tra2**β** is amplified in particular tumours including those of the lung, ovary, cervix, stomach, head, and neck. Both *TRA2B* RNA and Tra2**β** protein levels are upregulated in breast, cervical, ovarian, and colon cancer, and Tra2**β** expression is associated with cancer cell survival. The *TRA2B* gene is a transcriptional target of the protooncogene ETS-1 which might cause higher levels of expression in some cancer cells which express this transcription factor. Known Tra2**β** splicing targets have important roles in cancer cells, where they affect metastasis, proliferation, and cell survival. Tra2**β** protein is also known to interact directly with the RBMY protein which is implicated in liver cancer.

## 1. Introduction

Cancer is associated with a number of distinctive disease hallmarks [1]. These hallmarks include the ability of cancer cells to continuously divide by maintaining proliferative signalling pathways and to evade growth suppressors, to resist cell death; to induce angiogenesis to ensure a supply of oxygen and nutrition, and to invade other parts of the body (metastasis). These hallmarks of cancer cells occur against other changes including decreasing genome stability and inflammation [1]. 

Changes in splicing patterns in cancer cells compared to normal cells can contribute to each of these cancer hallmarks through effects on the expression patterns of important protein isoforms which regulate cell behaviour [2–4]. The splicing alterations which occur in cancer cells are partially due to changes in the activity and expression of core spliceosome components [5] and in the RNA binding proteins which regulate alternative exon inclusion [6]. Changes in the splicing environment in cancer cells might have therapeutic implications. Drugs which target the spliceosome are also being developed as potential therapies for treating cancer patients [7]. 

**Figure 1:**
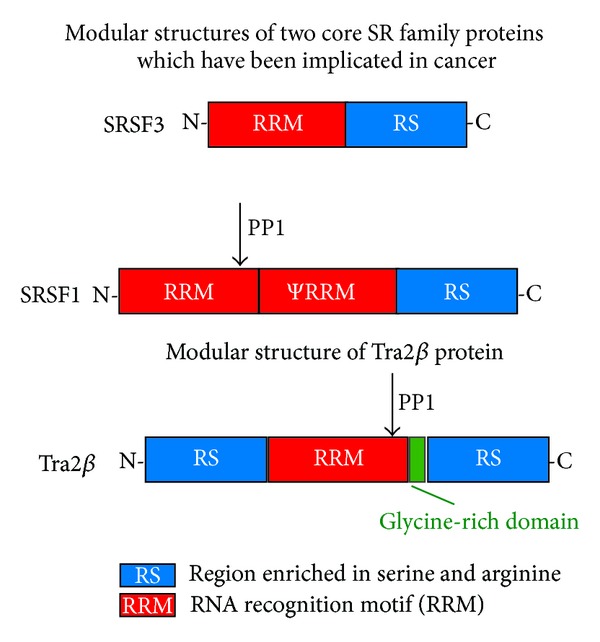
Modular structure of the core SR family proteins SRSF1 (also known as ASF/SF2) and SRSF3 (also known as SRp20) and the SR-like protein Tra2*β*. The RNA recognition motif (RRM) binds to target RNAs, and the RS region is responsible for protein-protein interactions. SRSF1 has a second RRM, annotated *ψ*RRM. SRSF1 and Tra2*β* have a PP1 docking site.

In this review, we particularly examine the potential role of the splicing regulator Tra2*β* as a modulator of gene function in cancer cells. Tra2*β* is part of a larger protein family which contains RNA recognition motifs (RRMs) and extended regions of serine and arginine residues (RS domains, named following the standard 1 letter amino acid code for serine and arginine) [8–10]. Core SR proteins include SRSF1 (previously known as ASF/SF2) and SRSF3 (previously known as SRP20) (Figure 1). Tra2*β* is considered an SR-like protein rather than a core SR family member because of two features. Firstly, Tra2*β* contains both an N- and C-terminal RS domains (each of the core members of the SR family has just a single C-terminal RS domain, with the RRM at the N-terminus). Secondly, the core group of SR proteins but not Tra2*β* can restore splicing activity to S100 extracts [11]. S100 extracts are made from lysed HeLa cells by high-speed ultracentrifugation to remove nuclei but contain most of the core spliceosome components necessary for splicing with the important exception of SR proteins which are insoluble in the magnesium concentrations used [12]. Addition of any single SR protein is sufficient to restore splicing activity to these S100 extracts [13].

Tra2*β* protein functions as a splicing regulator in the cell nucleus, where it activates the inclusion of alternative exons [14, 15]. Tra2*β* protein is able to interact with two types of RNA targets through its RRM. Firstly, the major RNA binding site for Tra2*β* is an AGAA-rich sequence [11, 16, 17]. Although an AGAA RNA sequence works best for Tra2*β* protein, an NGAA sequence is actually sufficient for binding. However, substituting the first A with either C, G, or T nucleotides in the NGAA target sequence decreases binding efficiency (the Kd value increases 2-fold between AGAA and NGAA) [16]. Secondly, the RRM of Tra2*β* is able to switch to a second mode of RNA binding, in which it interacts with single-stranded CAA-rich sequences within a stem loop structure [17]. 

When Tra2*β* binds to target RNA sites within an exon, it activates splicing inclusion of these bound exons into mRNA [11, 15–17]. Splicing activation by Tra2*β* protein is concentration dependent: increased Tra2*β* protein concentration leads to increased levels of target exon splicing inclusion [14, 15]. The RRMs of Tra2*β* and SRSF1 proteins both contain a docking site for protein phosphatase 1 (PP1), and dephosphorylation of these proteins by PP1 affects alternative splicing regulation [18].

Tra2*β* protein is encoded by the *TRA2B *gene (also called *SFRS10*) on human chromosome 3. As well as any potential role in cancer cells, Tra2*β* has important roles in normal development and is essential for normal mouse embryonic and brain development (*TRA2B* knockout mice fail to develop normally) [15, 19]. *TRA2B* has a paralog gene called* TRA2A* on the long arm of human chromosome 7, and this paralog encodes Tra2*α* protein [20]. Paralogs are additional copies of a gene derived by duplication. *TRA2A *derived by gene duplication from *TRA2B* early in the vertebrate lineage and so is found in all vertebrates. 

**Figure 2:**
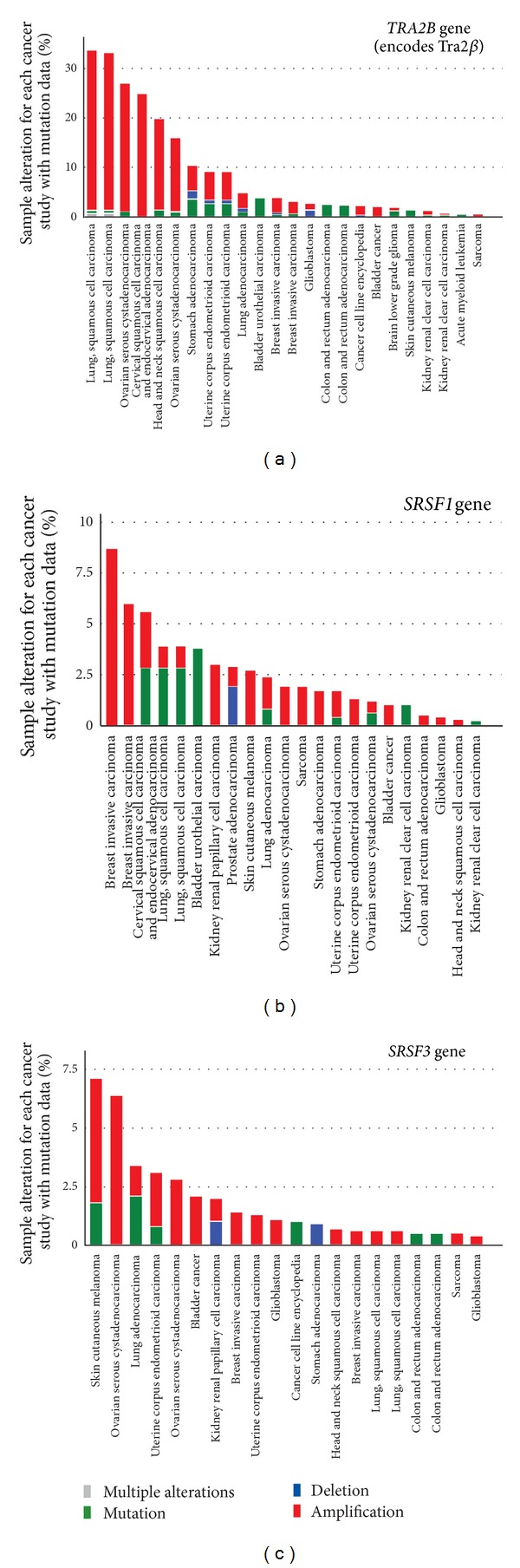
The (a) *TRA2B*, (b) *SRSF1* (also known as ASF/SF2), and (c) *SRSF3* (also known as SRP20) genes are amplified or otherwise mutated in several cancer types. For each of the three genes, data for genetic changes in all cancers were obtained using the cBioPortal database, filtering for percentage of altered cases (studies using mutation data) [22, 23]. The percentage, of cancer samples which showed genetic alterations in large cancer studies are shown on the *Y* axis and the respective type of cancer on the *X* axis. Full details of this kind of analysis are given on the cBioPortal website http://www.cbioportal.org/public-portal/index.do.

A number of the SR proteins have been found to have roles in cancer, amongst them, SRSF1 and SRSF3 (Figures 1 and 2). The mechanism of SRSF1 upregulation in cancer cells has been explained at a mechanistic level, and the effects of this upregulation in terms of gene expression control have been mapped onto the pathway of oncogenesis. Here, we review these important principles for SRSF1 and then apply these principles to gauge the likely effect of the Tra2*β* protein on cancer-specific gene expression.

## 2. SRSF1 Is Upregulated in Cancer and Is a Target for the Prooncogenic Transcription Factor Myc

SRSF1 upregulation in cancer cells can occur through two distinct mechanisms. Firstly, the *SRSF1* gene itself can become amplified in cancer. The *SRSF1* gene is on a region of chromosome 17q23 which is amplified in some breast cancers, including in tumours with a poor prognostic outlook and in the MCF7 breast cancer cell line [21]. Analysis of the *SRSF1* gene on the cBio Cancer Genomics Portal shows amplification of *SRSF1* mainly in breast cancers (Figure 2) [22, 23]. Secondly, *SRSF1* gene transcription is activated by the prooncogenic transcription factor Myc which is itself activated in some cancers. Myc upregulation in cancer leads to downstream increases in both *SRSF1* mRNA and SRSF1 protein expression [24].

Protein expression analysis using a highly specific monoclonal antibody showed that a number of tumours have increased SRSF1 protein compared to normal tissue [21]. As well as being upregulated in some cancer cells, *SRSF1* operates as a bona fide oncogene. Increased *SRSF1* gene expression can transform rodent fibroblasts in an NIH3T3 assay, and the resulting transformed cells form tumours in nude mice [21]. Tumour formation by these transformed fibroblasts is directly dependent on *SRSF1* expression, since it is blocked by parallel shRNA inhibition of *SRSF1* [21]. Together, these data suggest that upregulation of *SRSF1* gene expression can be one of the initial steps in oncogenesis. 

Experiments support an important function for SRSF1 protein in breast cancer cells. Mouse COMMA1-D mammary epithelial cells form tumours more efficiently in mice after transduction with *SRSF1*, and transduction of MF10A cells with *SRSF1* results in increased acinar size and decreased apoptosis in a 3D culture model [25]. A number of splicing targets have been identified which respond to increased levels of *SFRS1* expression in cancer cells (Table 1). These SRSF1-driven splicing changes produce prooncogenic mRNA splice isoforms, which encode proteins which decrease apoptosis and increase cellular survival and proliferation. 

## 3. Increased *SRSF3* Expression Is Also Associated with Cancer

Increased expression of the SR protein SRSF3 is also associated with cancer. The *SRSF3* gene is amplified in some cancers (Figure 2) [22, 23]. Loss of *SRSF3* expression in a number of cancer cell lines increases apoptosis and decreases proliferation, and increased expression of *SRSF3* leads to transformation of rodent fibroblasts and enables them to form tumours in nude mice [26]. 

Increased *SRSF3* expression levels have been associated with an increased tumour grade in ovarian cancer [27]. Intracellular levels of *SRSF3* mRNA are important for cancer cells: siRNA-mediated downregulation of *SRSF3* leads to cell cycle arrest at G1 in colon cancer cells, and their increased death through apoptosis. The mechanism of increased apoptosis in response to higher levels of SRSF3 protein might include aberrant splicing of the *HIPK2* pre-mRNA (which encodes an important apoptotic regulator related to *HIPK3*, which is a known splicing target of Tra2*β*), such that a proteasome-resistant form of HIPK2 protein is made after SRSF3 depletion [28].

## 4. Tra2***β*** Is Amplified in Particular Cancers and Is a Target of the Oncogenic Transcription Factor ETS-1

The *TRA2B* gene which encodes Tra2*β* becomes amplified in several cancers (Figure 2) and particularly in cancers of the lung, cervix, head and neck, ovary, stomach, and uterus [22, 23]. Upregulation of Tra2*β* protein expression has also been observed in several cancers, including breast, cervical and ovarian [29–31], and colon [32]. Tra2*β* upregulation is associated with invasive breast cancer [30], and medium to high Tra2*β* expression correlates with a poorer prognosis in cervical cancer compared to patients with lower expression levels [29]. 

Tra2*β* protein expression has been demonstrated to be important for cancer cell biology. Downregulation of Tra2*β* inhibits cell growth of a gastric cancer cell line, measured by a corresponding decrease in BrdU incorporation which monitors cells which have entered S phase [33]. Knockdown of Tra2*β* in colon cancer cells reduced cell viability and increased the level of apoptosis monitored using a TUNEL assay and through measurement of levels of cleaved PARP [32].

As well as *TRA2B* gene amplification, the expression levels of the ETS-1 transcription factor provide a possible mechanism through which Tra2*β* might be upregulated in cancer cells. Regulated transcription of the *TRA2B* gene in human colon cells is positively controlled by binding of the HSF1 and ETS-1 transcription factors to its promoter proximal region [32]. The ETS-1 protein is itself encoded by a protooncogene. ETS1 expression in metastatic breast cancer correlates with a poor prognosis [34, 35] and is associated with an invasive phenotype [36]. Expression of both ETS-1 [35] and Tra2*β* [37] might also be under control of estrogen, which is a key driver of estrogen receptor positive breast cancer development. Taken together, these observations suggest that the pathological mechanism of Tra2*β* upregulation in cancer cells might result from underlying changes in transcription factors in cancer cells. Other positive regulators of cell growth might also stimulate Tra2*β* expression, since expression of Tra2*β* is upregulated in response to growth factors in normal smooth muscle cells [38].

Reactive oxygen species made during inflammation provide a further potential mechanism for Tra2*β* upregulation in cancer cells. Tra2*β* expression is activated in response to reoxygenation of astrocytes following a period of oxygen deprivation and by ischaemia in rat brains [39]. Expression of Tra2*β* in smooth muscle cells is similarly induced following reoxygenation of hypoxic cells [38], and is upregulated in response to oxidative stress in human colorectal carcinoma cell line HCT116 [32]. Ischaemia has also been reported to induce cytoplasmic accumulation of Tra2*β* along with accompanying changes in splice site use [40]. Tra2*β* translocates into the cytoplasm in gastric cancer cells in response to cell stress induced by sodium arsenate [32], and changes in the nuclear concentration of Tra2*β* might have downstream effects on the splicing inclusion of target exons.

The increased levels of Tra2*β* observed in cancer cells mean that the *TRA2B* gene must be able to bypass the normal feedback expression control mechanisms which exist to keep Tra2*β* protein levels under tight control. An important feedback control mechanism uses an alternatively spliced “poison exon” in the *TRA2B* gene. Poison exons introduce premature stop codons when they are spliced into mRNAs, preventing translation of full-length proteins and often targeting mRNAs for nonsense-mediated decay [41]. Poison exon splicing into the *TRA2B* mRNA is activated by binding of Tra2*β* itself. Splicing inclusion of this poison exon acts as a brake on production of more Tra2*β* protein. The predicted outcome is that increased expression of Tra2*β* protein should lead to increased *TRA2B *poison exon inclusion and so correspondingly less newly translated Tra2*β* protein through a negative feedback loop [42]. 

Similarly, the levels of SRSF1 and the other SR proteins are thought to be normally autoregulated through poison exon inclusion [43]; so these other SR proteins must similarly bypass these mechanisms in cancer cells to enable their higher levels of expression to be established. 

## 5. Tra2***β*** Protein Regulates Splicing Patterns Which Are Important to Cancer Cells 

How might upregulation of Tra2*β* affect the biology of cancer cells? Three Tra2*β*-target exons have been identified in genes known to have important roles in cancer cells (Table 2). For two of these target exons, the actual regulated isoforms have also been demonstrated in cancer cells. 

Firstly, strong Tra2*β* binding to a cancer-associated exon in the *nuclear autoantigenic sperm protein* (abbreviated *NASP*) gene has been detected using HITS-CLIP of endogenous Tra2*β* protein in the mouse testis [14, 15]. This Tra2*β*-target exon is abbreviated* NASP-T*. Whilst the somatic *NASP* splice isoform is expressed ubiquitously, the *NASP-T* splicing isoform has a much tighter anatomic distribution and its splicing is associated particularly with cancer cells and embryonic development. While most normal adult tissues do not splice the *NASP-T *exons into their mRNAs, high levels of splicing inclusion are seen in the testis and to a lesser extent the heart, gut, and ovary [15].

**Figure 3:**
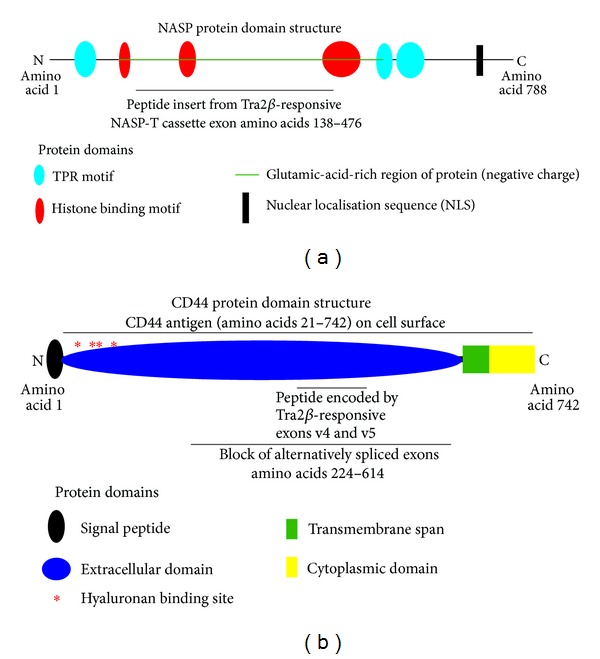
Protein domain architecture of known Tra2*β* splicing targets which are expressed in cancer cells. (a) Modular structure of NASP protein assembled from the UniProt database (http://www.uniprot.org/uniprot/P49321) [46], showing the position of the peptide insert encoded by the Tra2*β*-target exon NASP-T. (b) Modular structure of CD44 protein assembled using information from the UniProt database (http://www.uniprot.org/uniprot/P16070#P16070-6) [46], showing the position of peptide sequences encoded by the Tra2*β*-target exons CD44 v4 and v5. The CD44 antigen is displayed on the cell surface, and the protein is anchored on the cell surface by a single trans-membrane domain. Alternative isoforms are made through alternative splicing of 10 exons out of 19 encoding amino acids in the extracellular domain and also 2 exons which encode peptide sequence in the cytoplasmic domain. The two exons reported CD44 v4 and v5 exons correspond to amino acids 386–428 and 429–472, respectively, in the encoded protein. The protein domain structures are not drawn to scale.

Splicing inclusion of the *NASP-T* exon is strongly activated in transfected cells in response to coexpression of Tra2*β*, and *NASP-T* splicing also decreases in *TRA2B* knockout mouse brains compared to wild type, confirming that the *NASP-T* exon is a bona fide regulated target exon of Tra2*β* [14, 15]. Tra2*β* is currently the only known splicing regulator of the *NASP-T *exon. The *NASP-T* exon is unusually long (a 975 nucleotide long cassette exon, while the typical size for a human exon is more like 120 nucleotides), with at least 37 Tra2*β* protein binding sites within its sequence, making a very responsive target for Tra2*β* expression. Splicing inclusion of the *NASP-T* exon into the *NASP* mRNA introduces the coding information for an extra 375 amino acids into the encoded NASP protein (Figure 3). 

The NASP protein has a strongly biased peptide sequence which contains a high frequency of glutamic acid residues. The negative charges of the glutamic acid residues facilitate interactions with the positively charged histone partner proteins that NASP protein interacts with. NASP proteins also use tetratricopeptide repeats (TPRs) and histone binding motifs to facilitate interactions with protein partners including histones [44]. Both the somatic (sNASP) and NASP-T isoforms of the NASP protein contain the same TPRs involved in protein-protein interactions and seem to be functionally interchangeable in cells [45]. However, the longer NASP-T protein isoform has an additional histone binding motif and a longer stretch of the glutamic-acid-enriched sequence, suggesting that it might more efficiently interact with histones (Figure 3(a)). The NASP-T peptide cassette also adds a number of potentially phosphorylated serine and threonine residues to the NASP protein [44, 46]. Splicing inclusion of the *NASP-T* exon is likely to be important in cancer cells. The specific siRNA-mediated downregulation of *NASP* mRNAs containing the *NASP-T* exon leads to a block in proliferation and increased levels of apoptosis in cancer cells [47, 48].

Isoforms of the NASP protein with and without the peptide cassette inserted by the NASP-T exon are molecular chaperones which import histone H1 into the nucleus [49]. NASP protein isoforms also stably maintain the soluble pools of H3 and H4 histones needed for assembly of chromatin at times of high replication activity and are part of the complexes which load these into chromatin [45]. The *NASP* gene is critical for cell cycle progression in cultured cells and for mouse embryogenesis [50]. 

Why might NASP protein be important for cancer cells? *NASP *belongs to a network of genes important for cell survival [51], and NASP protein is a tumour-associated antigen in ovarian cancer [52]. NASP is highly expressed in S phase of the cell cycle [49], when chromatin needs to be reassembled after replication. Higher levels of NASP protein expression might be needed by cancer cells to enable their higher rates of replication to be achieved. NASP protein also has other roles related to chromatin stability. NASP protein is phosphorylated by the ATM and ATR kinases in response to ionising radiation and implicated in the repair of DNA double strand breaks [53]. One of the protein partners of NASP protein is the DNA repair protein Ku, and the yeast homologue of NASP is present at double strand breaks suggesting an important role in DNA repair (reviewed in [44]).

The second known splicing target of Tra2*β* with likely important functions in cancer cells is within the* CD44* pre-mRNA. *CD44 *encodes an important transmembrane protein partly displayed on the cell surface as the CD44 antigen (Figure 3(b)). CD44 protein acts as a receptor for hyaluronic acid and possibly other molecules and controls interactions with other cells, the extracellular matrix, and cellular motility through modulation of intracellular signalling cascades [54]. 

The N- and C-termini of the CD44 protein are encoded by constitutive exons, but the *CD44 *gene also contains an internal block of 10 consecutive internal alternative exons which are differentially regulated during development and in cancer [55]. These alternative exons encode portions of the extracellular domain of the protein (Figure 3(b)). *CD44* variable exons show variant splicing inclusion in breast cancer cells [30]. In particular, two *CD44* internal variable exons, *CD44*v4 and *CD44*v5, increase their splicing inclusion in transfected HeLa cells in response to increased Tra2*β* protein expression [30], suggesting that Tra2*β* might also increase their inclusion in breast tumours with elevated Tra2*β* expression. Although expression of variant CD44 exons has historically been associated with cancer metastasis, the picture regarding *CD44 *alternative splicing in cancer is complex. Very recent data suggest that the standard isoform of *CD44* mRNA (without splicing inclusion of its variable exons) might in fact play a key role in metastatic breast cancer, particularly in enabling an epithelial-mesenchyme transition of breast cancer cells [56].

The third known Tra2*β*-target exon which might be potentially relevant in cancer cells is in the *HIPK3* gene, which encodes a serine/threonine kinase involved in transcriptional regulation and negative control of apoptosis. High cellular levels of Tra2*β* stimulate splicing inclusion of a poison exon called HIPK3-T into the HIPK3 mRNA [57]. Normal HIPK3 protein is concentrated in subnuclear structures called promyelocytic leukemia bodies (PML bodies). The shorter HIPK3 protein isoform made under control of Tra2*β* fails to localise in PML bodies and lacks regions of the protein predicted to bind the androgen receptor, homeodomains, Fas, and p53 [57]. HIPK3-T is not confirmed as a splicing target of Tra2*β* in cancer, since splicing of the HIPK3-T exon has only been observed thus far in human testis and has not been directly reported from cancer cells [57]. 

## 6. Tra2***β*** Is Involved in Protein Interaction Networks with Partner Proteins Involved in Cancer

Some of the proteins which are known to interact either directly or indirectly with Tra2*β* have themselves been implicated with roles in cancer cells. Tra2*β* directly interacts with members of the hnRNP G family of proteins which includes the prototypic member hnRNP G (encoded by the *RBMX* gene located on the X chromosome); RBMY protein (which is encoded by a multigene family on the Y chromosome); and a number of retrogene-derived proteins. Of these retrogene-derived proteins, one called HNRNP G-T is both highly conserved in mammals and specifically expressed in meiosis. The interaction between Tra2*β* and hnRNP G family members likely buffers the splicing activity of Tra2*β* [58, 59], although they might also coregulate some target exons [60]. Expression of the RBMY protein has been directly implicated in liver cancer biology, where it may contribute to the male specificity of this cancer [61]. RBMY protein also interacts with SRSF3 protein [62]. 

## 7. Summary

**Figure 4:**
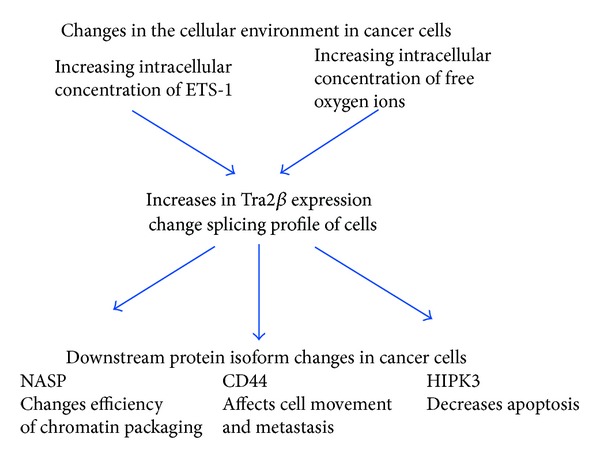
Hypothetical model suggesting how changes in the cellular environment may influence the expression of Tra2*β* and lead to downstream changes in mRNA splice isoform production.

The splicing regulator Tra2*β* is upregulated in some human cancers. Possible mechanisms for this upregulation include changes in oncogenic transcription factor expression and oxygen free radical concentrations in neoplastic tissue, both of which affect *TRA2B* gene expression (Figure 4). We do not currently know whether the *TRA2B *gene can function as an oncogene in its own right until experiments to test transformation of NIH3T3 cells are performed or the behaviour of such transformed cells in nude mice is tested. However, we do know that some of the known splicing targets of Tra2*β* identified in normal tissues are important for cancer cell biology and are particularly implicated in cell division and motility. Tra2*β* is essential during embryonic development, and many embryonic developmental pathways involved in cell growth and motility which are turned off in adult cells often become reactivated in cancer cells. Future analysis of the role of Tra2*β* in cancer cells will require the detailed identification of its endogenous splicing targets in cancer cells and the elucidation of their physiological roles. 

## Figures and Tables

**Table 1:** Known prooncogenic splicing targets of SFRS1 (previously known as ASF/SF2).

Splicing target	Possible role in cancer cells	Reference
*RON *	Δexon11 splice isoform increases cell motility and metastasis	[21, 25]
*BIN *	*BIN12a* splice isoform encodes protein no longer able to bind Myc and acts as tumour suppressor	[21, 25]
*MNK2 *	*MNK2* 13b splice isoform makes kinase which can phosphorylate EIF4E independent of MAP kinase activation	[21, 25]
*S6K *	Promotes oncogenic isoform of S6kinase which phosphorylates small subunit of ribosome	[21, 25]
*MCL-1/BCL-X/CASPASE9 *	Promotes production of antiapoptotic mRNAs to result in cell survival	[63–65]

**Table 2:** Known pro-oncogenic splicing targets of Tra2*β*.

Splicing target	Possible role in cancer cells	Reference
*CD44 *	Affects cancer cell mobility and metastasis	[30]
*Homeodomain-interacting kinase 3 (HipK3) *	*HIPK3* increases phosphorylation of cJun and cell proliferation	[57]
*Nasp-T *	Histone chaperone important for efficient replication Implicated in DNA repair processes	[15]

## References

[1] Hanahan D, Weinberg RA (2011). Hallmarks of cancer: the next generation. *Cell*.

[2] Rajan P, Elliott DJ, Robson CN, Leung HY (2009). Alternative splicing and biological heterogeneity in prostate cancer. *Nature Reviews Urology*.

[3] Venables JP (2004). Aberrant and alternative splicing in cancer. *Cancer Research*.

[4] Venables JP (2006). Unbalanced alternative splicing and its significance in cancer. *BioEssays*.

[5] Quesada V, Conde L, Villamor N (2012). Exome sequencing identifies recurrent mutations of the splicing factor SF3B1 gene in chronic lymphocytic leukemia. *Nature Genetics*.

[6] Grosso AR, Martins S, Carmo-Fonseca M (2008). The emerging role of splicing factors in cancer. *EMBO Reports*.

[7] Bonnal S, Vigevani L, Valcarcel J (2012). The spliceosome as a target of novel antitumour drugs. *Nature Reviews Drug Discovery*.

[8] Long JC, Caceres JF (2009). The SR protein family of splicing factors: master regulators of gene expression. *Biochemical Journal*.

[9] Shepard PJ, Hertel KJ (2009). The SR protein family. *Genome Biology*.

[10] Zhou Z, Fu XD (2013). Regulation of splicing by SR proteins and SR protein-specific kinases. *Chromosoma*.

[11] Tacke R, Tohyama M, Ogawa S, Manley JL (1998). Human Tra2 proteins are sequence-specific activators of pre-mRNA splicing. *Cell*.

[12] Krainer AR, Maniatis T (1985). Multiple factors including the small nuclear ribonucleoproteins U1 and U2 are necessary for Pre-mRNA splicing in vitro. *Cell*.

[13] Zahler AM, Neugebauer KM, Lane WS, Roth MB (1993). Distinct functions of SR proteins in alternative pre-mRNA splicing. *Science*.

[14] Elliott DJ, Best A, Dalgliesh C, Ehrmann I, Grellscheid S (2012). How does Tra2beta protein regulate tissue-specific RNA splicing?. *Biochemical Society Transactions*.

[15] Grellscheid S, Dalgliesh C, Storbeck M (2011). Identification of evolutionarily conserved exons as regulated targets for the splicing activator Tra2*β* in development. *PLoS Genetics*.

[16] Cléry A, Jayne S, Benderska N, Dominguez C, Stamm S, Allain FH-T (2011). Molecular basis of purine-rich RNA recognition by the human SR-like protein Tra2-*β*1. *Nature Structural and Molecular Biology*.

[17] Tsuda K, Someya T, Kuwasako K (2011). Structural basis for the dual RNA-recognition modes of human Tra2-*β* RRM. *Nucleic Acids Research*.

[18] Novoyatleva T, Heinrich B, Tang Y (2008). Protein phosphatase 1 binds to the RNA recognition motif of several splicing factors and regulates alternative pre-mRNA processing. *Human Molecular Genetics*.

[19] Mende Y, Jakubik M, Riessland M (2010). Deficiency of the splicing factor Sfrs10 results in early embryonic lethality in mice and has no impact on full-length SMN /Smn splicing. *Human Molecular Genetics*.

[20] Meyer LR, Zweig AS, Hinrichs AS (2013). The UCSC Genome Browser database: extensions and updates 2013. *Nucleic Acids Research*.

[21] Karni R, De Stanchina E, Lowe SW, Sinha R, Mu D, Krainer AR (2007). The gene encoding the splicing factor SF2/ASF is a proto-oncogene. *Nature Structural and Molecular Biology*.

[22] Cerami E, Gao J, Dogrusoz U (2012). The cBio cancer genomics portal: an open platform for exploring multidimensional cancer genomics data. *Cancer Discovery*.

[23] Gao J, Aksoy BA, Dogrusoz U (2013). Integrative analysis of complex cancer genomics and clinical profiles using the cBioPortal. *Science Signaling*.

[24] Das S, Anczukow O, Akerman M, Krainer AR (2012). Oncogenic splicing factor SRSF1 is a critical transcriptional target of MYC. *Cell Reports*.

[25] Anczuków O, Rosenberg AZ, Akerman M (2012). The splicing factor SRSF1 regulates apoptosis and proliferation to promote mammary epithelial cell transformation. *Nature Structural and Molecular Biology*.

[26] Jia R, Li C, McCoy JP, Deng C-X, Zheng Z-M (2010). SRp20 is a proto-oncogene critical for cell proliferation and tumor induction and maintenance. *International Journal of Biological Sciences*.

[27] He X, Arslan AD, Pool MD (2011). Knockdown of splicing factor SRp20 causes apoptosis in ovarian cancer cells and its expression is associated with malignancy of epithelial ovarian cancer. *Oncogene*.

[28] Kurokawa K, Akaike Y, Masuda K (2013). Downregulation of serine/arginine-rich splicing factor 3 induces G1 cell cycle arrest and apoptosis in colon cancer cells. *Oncogene*.

[29] Gabriel B, Hausen AZ, Bouda J (2009). Significance of nuclear hTra2-beta1 expression in cervical cancer. *Acta Obstetricia et Gynecologica Scandinavica*.

[30] Watermann DO, Tang Y, Hausen AZ, Jäger M, Stamm S, Stickeler E (2006). Splicing factor Tra2-*β*1 is specifically induced in breast cancer and regulates alternative splicing of the CD44 gene. *Cancer Research*.

[31] Fischer D-C, Noack K, Runnebaum IB (2004). Expression of splicing factors in human ovarian cancer. *Oncology Reports*.

[32] Kajita K, Kuwano Y, Kitamura N (2013). Ets1 and heat shock factor 1 regulate transcription of the *Transformer* 2*β* gene in human colon cancer cells. *Journal of Gastroenterology*.

[33] Takeo K, Kawai T, Nishida K (2009). Oxidative stress-induced alternative splicing of transformer 2*β* (SFRS10) and CD44 pre-mRNAs in gastric epithelial cells. *American Journal of Physiology*.

[34] Lincoln DW, Bove K (2005). The transcription factor Ets-1 in breast cancer. *Frontiers in Bioscience*.

[35] Lincoln DW, Phillips PG, Bove K (2003). Estrogen-induced Ets-1 promotes capillary formation in an in vitro tumor angiogenesis model. *Breast Cancer Research and Treatment*.

[36] Dittmer J (2003). The biology of the Ets1 proto-oncogene. *Molecular Cancer*.

[37] Zhang X, Moor AN, Merkler KA, Liu Q, McLean MP (2007). Regulation of alternative splicing of liver scavenger receptor class B gene by estrogen and the involved regulatory splicing factors. *Endocrinology*.

[38] Tsukamoto Y, Matsuo N, Ozawa K (2001). Expression of a novel RNA-splicing factor, RA301/Tra2*β*, in vascular lesions and its role in smooth muscle cell proliferation. *American Journal of Pathology*.

[39] Matsuo N, Ogawa S, Imai Y (1995). Cloning of a novel RNA binding polypeptide (RA301) induced by hypoxia/reoxygenation. *Journal of Biological Chemistry*.

[40] Daoud R, Mies G, Smialowska A, Oláh L, Hossmann K-A, Stamm S (2002). Ischemia induces a translocation of the splicing factor tra2-*β*1 and changes alternative splicing patterns in the brain. *Journal of Neuroscience*.

[41] McGlincy NJ, Smith CWJ (2008). Alternative splicing resulting in nonsense-mediated mRNA decay: what is the meaning of nonsense?. *Trends in Biochemical Sciences*.

[42] Stoilov P, Dauod R, Nayler O, Stamm S (2004). Human tra2-beta1 autoregulates its protein concentration by infuencing alternative splicing of its pre-mRNA. *Human Molecular Genetics*.

[43] Lareau LF, Inada M, Green RE, Wengrod JC, Brenner SE (2007). Unproductive splicing of SR genes associated with highly conserved and ultraconserved DNA elements. *Nature*.

[44] Finn RM, Ellard K, Eirin-Lopez JM, Ausio J (2012). Vertebrate nucleoplasmin and NASP: egg histone storage proteins with multiple chaperone activities. *The FASEB Journal*.

[45] Cook AJL, Gurard-Levin ZA, Vassias I, Almouzni G (2011). A specific function for the histone chaperone NASP to fine-tune a reservoir of soluble H3-H4 in the histone supply chain. *Molecular Cell*.

[46] UniProt Consortium (2012). Reorganizing the protein space at the Universal Protein Resource (UniProt). *Nucleic Acids Research*.

[47] Alekseev OM, Richardson RT, Tsuruta JK, O’Rand MG (2011). Depletion of the histone chaperone tNASP inhibits proliferation and induces apoptosis in prostate cancer PC-3 cells. *Reproductive Biology and Endocrinology*.

[48] Ma W, Xie S, Ni M (2012). MicroRNA-29a inhibited epididymal epithelial cell proliferation by targeting nuclear autoantigenic sperm protein (NASP). *Journal of Biological Chemistry*.

[49] Richardson RT, Batova IN, Widgren EE (2000). Characterization of the histone H1-binding protein, NASP, as a cell cycle-regulated somatic protein. *Journal of Biological Chemistry*.

[50] Richardson RT, Alekseev OM, Grossman G (2006). Nuclear autoantigenic sperm protein (NASP), a linker histone chaperone that is required for cell proliferation. *Journal of Biological Chemistry*.

[51] Alekseev OM, Richardson RT, Alekseev O, O’Rand MG (2009). Analysis of gene expression profiles in HeLa cells in response to overexpression or siRNA-mediated depletion of NASP. *Reproductive Biology and Endocrinology*.

[52] Ali-Fehmi R, Chatterjee M, Ionan A (2009). Analysis of the expression of human tumor antigens in ovarian cancer tissues. *Cancer Biomarkers*.

[53] Matsuoka S, Ballif BA, Smogorzewska A (2007). ATM and ATR substrate analysis reveals extensive protein networks responsive to DNA damage. *Science*.

[54] Ponta H, Sherman L, Herrlich PA (2003). CD44: from adhesion molecules to signalling regulators. *Nature Reviews Molecular Cell Biology*.

[55] Screaton GR, Bell MV, Jackson DG, Cornelis FB, Gerth U, Bell JI (1992). Genomic structure of DNA encoding the lymphocyte homing receptor CD44 reveals at least 12 alternatively spliced exons. *Proceedings of the National Academy of Sciences of the United States of America*.

[56] Brown RL, Reinke LM, Damerow MS (2011). CD44 splice isoform switching in human and mouse epithelium is essential for epithelial-mesenchymal transition and breast cancer progression. *Journal of Clinical Investigation*.

[57] Venables JP, Bourgeois CF, Dalgliesh C, Kister L, Stevenin J, Elliott DJ (2005). Up-regulation of the ubiquitous alternative splicing factor Tra2*β* causes inclusion of a germ cell-specific exon. *Human Molecular Genetics*.

[58] Liu Y, Bourgeois CF, Pang S (2009). The germ cell nuclear proteins hnRNP G-T and RBMY activate a testis-specific exon. *PLoS Genetics*.

[59] Nasim MT, Chernova TK, Chowdhury HM, Yue B-G, Eperon IC (2003). HnRNP G and Tra2*β*: opposite effects on splicing matched by antagonism in RNA binding. *Human Molecular Genetics*.

[60] Hofmann Y, Lorson CL, Stamm S, Androphy EJ, Wirth B (2000). Htra2-*β*1 stimulates an exonic splicing enhancer and can restore full-length SMN expression to survival motor neuron 2 (SMN2). *Proceedings of the National Academy of Sciences of the United States of America*.

[61] Tsuei D-J, Lee P-H, Peng H-Y (2011). Male germ cell-specific RNA binding protein RBMY: a new oncogene explaining male predominance in liver cancer. *PLoS ONE*.

[62] Elliott DJ, Bourgeois CF, Klink A, Stévenin J, Cooke HJ (2000). A mammalian germ cell-specific RNA-binding protein interacts with ubiquitously expressed proteins involved in splice site selection. *Proceedings of the National Academy of Sciences of the United States of America*.

[63] Moore MJ, Wang Q, Kennedy CJ, Silver PA (2010). An alternative splicing network links cell-cycle control to apoptosis. *Cell*.

[64] Paronetto MP, Achsel T, Massiello A, Chalfant CE, Sette C (2007). The RNA-binding protein Sam68 modulates the alternative splicing of Bcl-x. *Journal of Cell Biology*.

[65] Gautrey HL, Tyson-Capper AJ (2012). Regulation of Mcl-1 by SRSF1 and SRSF5 in cancer cells. *PLoS ONE*.

